# Clinical Ratings of White Matter Hyperintensities, Hippocampal Ratings, and Neuropsychological Functioning from The Cache County Memory Study

**DOI:** 10.14283/jarlife.2022.2

**Published:** 2022-03-21

**Authors:** T.J. Farrer, E.D. Bigler, Y.H.W. Tsui-Caldwell, T.J. Abildskov, J.A.T. Tschanz, M.C. Norton, K.A. Welsh-Bohmer

**Affiliations:** 1 Department of Psychology, Brigham Young University, Provo, UT, USA; 2 Neuroscience Center, Brigham Young University, Provo, UT, USA; 3 University of Utah, Salt Lake City, UT, USA; 4 Department of Psychology, Utah State University, Logan, UT, USA; 5 Center for Epidemiologic Studies, Utah State University, Logan, UT, USA; 6 Department of Family, Consumer & Human Development, Utah State University, Logan, UT, USA; 7 Department of Psychiatry and Neurology, Duke University, Durham, NC

**Keywords:** Scheltens Rating Scale, Cache County, aging, clinical ratings;, white matter hyperintensity, memory

## Abstract

**Objective:**

White matter burden and medial temporal atrophy are associated with cognitive health. A large epidemiological database, such as the Cache County Memory Study (CCMS), can provide additional insight into how visual clinical ratings of brain structural integrity predict cognition in older adults.

**Method:**

We used the Scheltens Ratings Scale to quantify white matter lesion burden and medial temporal atrophy in the CCMS sample to determine if these qualitative markers are predictive of memory function. We performed clinical ratings of MRI scans across two ascertainment periods among 187 community-dwelling older adults and correlated these ratings with MMSE, CERAD memory performance, and general cognitive ability.

**Results:**

Higher Scheltens ratings measuring white matter and basal ganglia hyperintensities were associated with lower memory performance (r = 0.21). The strongest correlations were observed between medial temporal atrophy and general cognition performance (r = 0.32).

**Conclusions:**

The current findings support previous research that the integrity of different regions of the brain correlate to function in a meaningful way.

## Introduction

In 1994, a population-based study of aging and memory was conducted in Cache County, Utah (1), hereby referred to as the Cache County Memory Study (CCMS). This study involved collecting clinical assessments and neuroimaging data from approximately 6,000 individuals with suspected dementia or other cognitive or neuropsychiatric disorders at three ascertainments, referred to as “Wave 1,” “Wave 2,” and “Wave 3.” As part of the clinical assessment, all subjects were administered the Modified Mini-Mental Status Exam (2) at screening and a more detailed clinical evaluation that included measures derived from the Consortium to Establish a Registry for Alzheimer’s Disease (CERAD) battery (3) and other tests to improve sensitivity and specificity of case detection (4).

The initial ascertainment, Wave 1, began in 1995 and ran through 1997. All participants from this Wave were scanned by a 0.5 Tesla magnetic resonance imaging (MRI) scanner. Approximately three years later, the MRI scanner was upgraded to 1.5 Tesla. As a result, all participants from Wave 2 and onward were scanned by the upgraded field strength. Despite the utilization of two different field strengths, Scheltens Rating Scales were shown to be reliable in detecting white matter (WM) burden in both normal aging and dementing samples (5).

Clinical ratings derived from MRI studies provide some level of objectivity beyond that of clinical judgment alone. Clinical ratings can be assessed quickly and efficiently and can usually be completed on scans that may differ in the acquisition sequence and parameters used to obtain the image. Furthermore, having a standard against which the ratings are made permits both the clinician and researcher to use common metrics to report findings.

One of the most common clinical rating schemas for assessing white matter hyperintensities (WMHs) and hippocampal atrophy is the Scheltens Rating Methods (1, 6). To obtain a WMHs rating, either the proton-density (PD) or fluid attenuation inversion recovery (FLAIR) sequence are used to identify bright-white signal abnormalities within both white matter and gray matter regions of the brain. Hippocampal atrophy is determined by visual inspection of hippocampal size, as well as size of the choroidal fissure and temporal horn. These latter two measures are not necessarily specific to hippocampal atrophy, but as the hippocampus loses volume there is a compensatory dilation of the temporal horn and a widening of the choroidal fissure. Temporal horn enlargement and widening of the choroidal fissure may also reflect more generalized volume loss of the temporal lobe and, as such, these measures may reflect overall integrity of the temporal lobe.

A basic assumption in neuropsychological assessment is that testing of memory function taps abilities dependent on temporal lobe integrity, in particular the hippocampus. In the current study, we descriptively explore the association between the Scheltens Rating Scales and memory assessment derived from the CERAD measures (3) with the hypothesis that white matter and medial temporal integrity are predictive of cognitive test performance. We also aim to provide incremental validity to other research utilizing clinical visual ratings as predictors of cognitive function in older adults.

## Method

Study procedures were approved by IRB committees at Duke University, Johns Hopkins University, and Utah State University at the time of original study enrollment. Archival analysis for the present study was approved by IRB at Brigham Young University.

### Participants

Three hundred and fifty two individuals (165 males [46.8%]) were drawn from the CCMS (1). This includes individual from Wave 2 of the CCMS of whom the noted data points were collected. Both males and females had similar age, age ranges, and educational levels in the current study. The mean age was 89.9 for females and 88 for males. Mean years of education was 12.7 for females and 14.2 for males. Mean MMSE scores were 22.7 for females and 23.8 for males. Additional detailed descriptions and clinical characteristics of the subjects have been published elsewhere (7, 8).

### MRI acquisition

All subjects underwent MRI with Wave 1 participants scanned on a 0.5 Tesla Phillips MRI scanner while Wave 2 participants were analyzed on a 1.5 Tesla Siemens MRI scanner. Imaging at 0.5 Tesla utilized a quadrature head coil with the following sequence details: T1-weighted sequence (TR (ms) 500 and TE of 15) with 2 excitations with an acquisition matrix of 256 X 256 with field of view (FOV) at 24 cm, slice thickness of 5mm and gap at 1 mm; axial T2-weighted and proton density imaging (PDI) sequences (TR (ms) 3148, TE of 31) with 90/1 excitations. Acquisition matrix was 256 X 256 with a FOV of 22 cm, slice thickness of 5mm and a gap of 1.5 mm; coronal dual spin-echo sequence (TR (ms) 3046 and a TE of 30) with 90/1 excitations. Acquisition matrix was 256 X 240 and FOV of 22 cm, slice thickness of 3mm and a gap of 0.3mm.

The 1.5 Tesla scans were obtained using the circular polarized array head coil with the following sequence parameters: Sagittal 1.5 Tesla scans were acquired using a T1 sequence (TR (ms) 500, TE of 14) with 1 excitation with and acquisition matrix of 256 X 192 with a FOV of 22 cm, slice thickness of 5mm, and gap at 1.5 mm. Flip angle was 90; T2, PDI and FLAIR sequences were all obtained in the axial plane, with the T2 sequence (TR (ms) of 6940, TE of 119) with 2 excitations and an acquisition matrix was 512 X 237 with a FOV of 17.5 X 22 cm, slice thickness of 4 mm, and gap at 0.8mm. Flip angle was 170; the PDI sequence (TR (ms) of 3000, TE of 17) with 1 excitation and an acquisition matrix of 256 X 202 with a FOV of 17.9 X 22 cm, slice thickness of 4.5mm, gap at 2 mm and a flip angle of 170; the FLAIR sequence (TR (ms) of 9000, TE of 104) with 1 excitation with an acquisition matrix of 256 X 163 with a FOV of 17.5 X 22 cm, slice thickness of 5mm, gap at 2 mm and flip angle of 170; coronal T2-weighted scans (TR (ms) 6940, TE of 119) with 2 excitations with an acquisition matrix of 512 X 237 with a FOV of 16.7 X 21 cm, slice thickness of 5 mm, gap at 1 mm and a flip angle of 170. For the Wave 1 Scheltens ratings were performed using the T2 and PD sequences. For Wave 2 ratings were based on T2 and FLAIR.

## Procedure

### Scheltens Rating Methods

Detailed procedures of the Scheltens Rating Methods for both WMHs and medial temporal lobe atrophy (MTA) have been published elsewhere (1, 9, 6). Briefly, the WMHs rating has four sum scores which consists of periventricular hyperintensities (PVH), lobar WMH, basal ganglia hyperintensities (BGH), and infratentorial foci of hyperintensity (IFH). Except for PVH, which were rated from 0-3, all other scores were rated from 0-6, with higher scores representing greater degree of WMH. Using the Scheltens Rating Methods in measuring MTA, three regions of interest (ROIs) were examined: temporal horn width, choroid fissure and hippocampal height. Head and body size differences were adjusted for by an intracranial width measurement.

### Cognitive Variables

Detailed description of the CERAD neuropsychological battery can be found in Welsh et al. (1994). Overall, the CERAD battery includes measures of mental status (MMSE), language, memory, and constructional praxis. In order to examine cognitive impairments not covered by the original CERAD battery, additional common neuropsychological tests were added in the battery to enhance the assessment of language, processing speed, overall intelligence, immediate and delayed memory (10, 4). The current study focused on the following two memory tests: Logical Memory I of the Wechsler Memory Scale and the Word List Memory Test from the CERAD.

For the purpose of this study, we also generated two composite scores based on cognitive functioning, including a General Memory Score and a General Cognition Score. The General Memory Score was derived by averaging the z scores of all memory tests. The General Cognition Score was calculated by first obtaining an average z score for each domain, including memory, and then averaging these z scores for an overall composite.

### Statistical Analysis

This study includes basic descriptive data in Table 1. Pearson Product Moment Correlation (r) analysis were used to assess the association between cognitive outcomes and clinical ratings of brain imaging. Alpha was set to p ≤ .05.

**Table 1:** Mean and Standard Deviation of Mini-Mental Status Exam and the Scheltens Ratings for White Matter Hyperintensity and Medial Temporal Lobe Atrophy

	Var	N	Mean	SD	Minimum	Maximum
Scheltens Ratings	MMSE (Total Score)	352	23.18	5.39	2.00	30.00
White Matter Hyperintensity	WMH	352	10.78	7.15	0.00	30.00
	BGH	352	1.60	2.73	0.00	18.00
	IFH	352	0.93	1.64	0.00	10.00
	PVM	352	6.23	2.16	0.00	9.00
	Scheltens Total	352	19.54	10.93	0.00	55.00
Medial Temporal Lobe Atrophy	Intracranial Width	351	120.12	9.64	101.10	149.77
	L Choroid Fissue	350	3.87	1.65	0.31	10.82
	R Choroid Fissue	350	3.87	1.69	0.30	12.24
	Choroid Fissue (Total)	350	7.75	3.03	0.81	21.09
	L Temporal Horn	350	5.89	3.28	0.19	21.80
	R Temporal Horn	350	6.24	3.90	0.16	43.35
	Temporal Horn (Total)	350	12.14	6.62	0.71	57.35
	L Hippocampus	350	12.97	2.17	0.83	18.24
	R Hippocampus	350	12.99	2.24	0.62	18.39
	Hippocampus (Total)	350	25.97	4.14	1.45	36.31

Note: Var = Variables; SD = Standard deviation; MMSE = Mini-Mental Status Exam; WMH = White matter hyperintensity; BGH = Basal ganglia hyperintensity; IFH = Infratentorial foci hyperintensity; PVH = Periventricular hyperintensity; L = Left; R = Right.

## Results

Table 1 summarizes overall mental status orientation and Scheltens Ratings for both WMHs and MTA for all subjects. Performances in memory tasks were as follows: Logical Memory (Mean = 11.28, SD = 6.92) and Word List Memory Task (Mean = 12.44, SD = 4.85).

Table 2 summarizes the correlation coefficients, including MMSE score, WMHs and MTA in this CCMS sample in relation to Logical Memory I of the Wechsler Memory Scale and Word List Memory Task derived from the CERAD (3) measures. Significant positive correlations were observed between MMSE and Logical Memory I (r = .56, p < .05) and Word List Memory Task (r = .65, p < .05), which suggests that higher overall cognitive ability was associated with better memory performance. Higher Scheltens Ratings in WMH, BGH, and total score, indicating increased levels of WM pathology, were associated with lower performance in both memory tasks. Hippocampus integrity was significantly and positively related to performance on both memory tasks, indicating that less hippocampal atrophy was associated with better memory performance. We did not find any relationship between clinical ratings of temporal horn dilation and memory tasks performance. Taken together, WMHs in lobar and basal ganglia were associated with lower memory tasks performance and general cognition. Furthermore, hippocampal integrity was related to higher memory tasks performance.

**Table 2:** Correlations between Memory Performance, Mini-Mental Status Exam and Scheltens Ratings

	MMSE	WMH	BGH	Scheltens Total	Temporal Horn (Total)	Hippocampus (Total)
Logical Memory	0.56*	-0.11*	-0.14*	-0.16*	-0.08	0.29*
Word List Memory Task	0.65*	-0.16*	-0.22*	-0.21*	-0.08	0.26*
General Memory Score	0.67*	-0.15*	-0.19*	-0.20*	-0.09	0.30*
General Cognition Score	0.84*	-0.19*	-0.21*	-0.24*	-0.09	0.32*

Note: MMSE = Mini-Mental Status Exam (total score); WMH = White matter hyperintensity; BGH = Basal ganglia hyperintensity. * p ≤ .05.

## Discussion

General white matter integrity and medial temporal atrophy are venerated anatomical correlates of cognition. We used archival data from the CCMS to further examine the association between visual ratings of the brain and memory in a large sample of older adults. As noted, memory performance and general cognition are related to both white matter and basal ganglia hyperintensities but the strongest correlations observed in this analysis were between cognitive function and total hippocampal volume. Clinical ratings of the temporal horn were not predictive of cognitive function. The findings support previous research that the integrity of different regions of the brain correlate to function in a meaningful way. While white matter and basal ganglia are important for a broad range of cognitive and executive processes, the medial temporal lobe and hippocampus are particularly critical for memory processing and general cognitive ability. These findings are similar to recent work by our group (5) in which we demonstrate that higher Scheltens Ratings are associated with lower MMSE performance in the CCMS sample. The current study and our previous work with this sample are important because of the role that structural brain integrity plays in the process of normal aging and dementia (11).

Other studies have used Scheltens Ratings to examine cognitive outcomes. Brickman et al. (12) demonstrated that white matter and general cerebral volume predict cognitive function and that baseline WMHs are associated with a faster rate of cognitive decline. Other studies subsequently demonstrated that Scheltens Ratings predict conversion from mild cognitive impairment (MCI) to Alzheimer’s disease (AD) and support a vascular component to AD pathology (13; 14). More recently, Smith et al. (15) also used Scheltens Ratings and other clinical ratings to demonstrate that white matter integrity predicts diagnostic groups status between AD and vascular dementia. Visual rating scores of vascular burden predict that those with MCI will progress to dementia 1.6-2.0 years faster and deep white matter lesions result a 3.5 to 3.8-fold increased risk in progression to dementia (16). Despite strong preexisting evidence that structural brain integrity is associated with cognition, we propose that the current findings represent an important addition to the literature. Specifically, structural brain integrity, whether measured via clinical ratings or by computer automation, does not always predict cognitive impairment or diagnosis of dementia. In fact, Claus et al. (17) recently examined the relative importance of white matter lesions and medial temporal atrophy on cognitive function in older adults. Also using Scheltens scale scores, these authors reported that white matter lesions predict both memory and non-memory performances (non-memory referred to language, attention, visual-perceptual, and executive functions). However, when controlling for MTA, white matter lesions continued to predict non-memory performance but no longer impacted memory scores. They also reported that white matter integrity negatively impacted non-memory function only when MTA was present. Taken together, Claus and colleagues suggest caution when using white matter integrity only in predicting cognitive outcomes.

There are limitations to the current work. The current study used different field strengths and different MRI platforms across the study period. The original 0.5 and 1.5 Tesla scans were not amenable to automated analysis of white matter or brain volume volumetric. However, clinical ratings are cost effective and easily applied to MR scans. We also previously demonstrated that clinical ratings are generally equivalent across field strengths in the CCMS sample (5). We suggest that the current findings support the use of qualitative analysis of brain health in large population-based samples. Our findings also support the utility of Scheltens Ratings and further support the fact that white matter and MTA predict memory function in older adults. This is consistent with a recent review by Prins and Scheltens (11) that clinical ratings should continue to be used to investigate how WMH, MTA, and dementia are related.

We conclude that both white matter lesion burden and MTA contribute to cognitive functioning in older community-dwelling adults, with the strongest associations between MTA and memory. These findings are important because they lend support to existing literature in a large epidemiologic sample of older adults and provide evidence that visual clinical ratings are a valid means of assessing structural brain integrity and predicting cognitive outcomes. Furthermore, information about clinical ratings and structural brain integrity from neuroimaging can be integrated with neuropsychological assessment findings, potentially enhancing the clinical significance of both measures (18). One without the other may be insufficient to make an accurate diagnosis. As noted in a recent large study of 824 older adults, dementia is frequently misclassified when based solely on cognitive assessment (35% misclassification rate; (19). In a clinical setting where shared decision making is increasingly becoming the norm, improved disease classification based on cognitive testing and imaging may be valuable to both patient and doctor (20).
